# The feedback loop between calcineurin, calmodulin-dependent protein kinase II, and nuclear factor of activated T-cells regulates the number of GABAergic neurons during planarian head regeneration

**DOI:** 10.3389/fnmol.2022.988803

**Published:** 2022-09-12

**Authors:** Hui Zhen, Mingyue Zheng, Huazhi Geng, Qian Song, Lili Gao, Zuoqing Yuan, Hongkuan Deng, Qiuxiang Pang, Bosheng Zhao

**Affiliations:** ^1^Laboratory of Developmental and Evolutionary Biology, Shandong University of Technology, Nantong, China; ^2^Guangdong Provincial People’s Hospital, Guangdong Academy of Medical Sciences, Guangzhou, China; ^3^Zibo Maternal and Child Health Hospital, Zibo, China

**Keywords:** *Caln*, *CamkII*, *NFAT*, GABAergic neurons, planarian, regeneration

## Abstract

Disturbances in the excitatory/inhibitory balance of brain neural circuits are the main source of encephalopathy during neurodevelopment. Changes in the function of neural circuits can lead to depolarization or repeat rhythmic firing of neurons in a manner similar to epilepsy. GABAergic neurons are inhibitory neurons found in all the main domains of the CNS. Previous studies suggested that *DjCamkII* and *DjCaln* play a crucial role in the regulation of GABAergic neurons during planarian regeneration. However, the mechanisms behind the regeneration of GABAergic neurons have not been fully explained. Herein, we demonstrated that DjCamkII and DjCaln were mutual negative regulation during planarian head regeneration. *DjNFAT* exerted feedback positive regulation on both *DjCaln* and *DjCamkII*. Whole-mount *in situ* hybridization (WISH) and fluorescence *in situ* hybridization (FISH) revealed that *DjNFAT* was predominantly expressed in the pharynx and parenchymal cells in intact planarian. Interestingly, during planarian head regeneration, *DjNFAT* was predominantly located in the newborn brain. Down-regulation of *DjNFAT* led to regeneration defects in the brain including regenerative brain became small and the lateral nerves cannot be regenerated completely, and a decreasein the number of GABAergic neurons during planarian head regeneration. These findings suggest that the feedback loop between *DjCaln, DjCamkII*, and *DjNFAT* is crucial for the formation of GABAergic neurons during planarian head regeneration.

## Introduction

Disturbances in the excitatory/inhibitory balance of brain neural circuits are the main source of encephalopathy during neurodevelopment. Once the function of neural circuits changes, the neurons in the corresponding circuits will depolarize or repeatedly fire rhythmically, which is a pattern closely related to the occurrence of epilepsy. γ-Aminobutyric acid (GABA) is the major inhibitory neurotransmitter in the central nervous system (CNS) and thereby plays a crucial role in the balance between inhibitory and excitatory neural circuits (Watanabe et al., 2002). GABAergic neurons are produced in all the main domains of the CNS, where they develop from discrete regions of the neuroepithelium. Therefore, it is expected that dysfunctions in the GABAergic system lead to neurodegenerative diseases, such as Parkinson’s disease (PD) and Huntington’s disease (HD) (Kleppner and Tobin, 2001; Galvan and Wichmann, 2007; Gajcy et al., 2010). Therefore, a certain degree of GABAergic neuron regeneration is essential to maintain the excitatory/inhibitory balance. In addition, it is widely accepted that continuous neurogenesis takes place in the adult hippocampus throughout the life of mammals, including humans (Eriksson et al., 1998; Zhao et al., 2008). The newborn neurons mostly differentiate into excitatory granular cells and functionally integrate into the preexisting hippocampal neural circuitry (Cameron and McKay, 2001; van Praag et al., 2002; Toni et al., 2007). However, little is known about the number of newborn cells that can regenerate and differentiate into inhibitory GABAergic interneurons in the damaged brain.

Calcineurin (Caln), also called protein phosphatase B (PP2B), is a Ca^2+^/calmodulin-dependent serine/threonine phosphatase that was first described in a bovine brain 40 years ago (Ho et al., 1976; Wang and Desai, 1976). The protein has a 60 kDa catalytic subunit (Caln A) and a 19 kDa regulatory subunit (Caln B) (Rusnak and Mertz, 2000; Li et al., 2011). Caln is located in the brain abundantly (Goto et al., 1986; Kuno et al., 1992). In neurons, Caln is localized in multiple organelles, such as cytoplasm, endoplasmic reticulum, and Golgi apparatus (Bram et al., 1993; Mehta and Zhang, 2014; Cardenas and Marengo, 2016). Studies indicate that GABA is regulated by Caln in synaptogenesis, for the reorganization and development of an adult CNS (Hayama et al., 2013; Toyoda et al., 2015; Wang et al., 2015).

Nuclear factor of activated T-cells (NFAT) can be dephosphorylated by Caln in order to translocate into the nucleus and become activated (Shaw et al., 1988; Schulz and Yutzey, 2004; Boothby, 2010). In the peripheral nervous system, Caln/NFAT signaling plays a critical role in the survival, proliferation, and differentiation of neural and glial precursor cells, especially during tissue regeneration (Serrano-Perez et al., 2015). Moreover, the neurotrophin nerve growth factor (NGF) regulates the up-regulation of the plasminogen activation system and synaptic protein plasminogen activator inhibitor 1 (PAI-1) in primary mouse hippocampal neurons through Caln/NFAT (Stefos et al., 2013). In mice, GABA_*A*_ receptors promote anxiety behavior and hippocampal neurogenesis through Caln/NFAT4, indicating that this signaling pathway may serve as a potential drug target for the treatment of mood disorders (Quadrato et al., 2014). Meanwhile, the transcription factor NFAT5 is expressed in the anterior blastema of tail fragments after 3 days into regeneration in planarian *Schmidtea mediterranea* (Suzuki-Horiuchi et al., 2021).

In human T Cells, activation of Ca^2+^/calmodulin-dependent protein kinase II (CamKII) can reduce the efficiency of NFAT by nearly 35% (Hama et al., 1995). In cardiomyocytes, the activation of cytoplasmic CamKII inhibits calmodulin and NFAT through phosphorylation (MacDonnell et al., 2009). In budding yeast, a lack of the CamKII homologue CMK2 increased the level of calcium/calcineurin signaling, as well as the expression levels of PMR1 and PMC1. The expression of target genes including *CMK2, RCN1, PMR1*, and *PMC1* is dependent on the activation of Crz1 (Mehta et al., 2009; Xu et al., 2019). These results indicate that CamKII is a negative feedback controller of the Caln/NFAT signaling pathway. However, whether there is a mutual regulation relationship between CamKII, Caln and NFAT or not in the neurons regeneration process has not been previously reported.

Planarians are often used as model organisms in the study of CNS regeneration due to their powerful regeneration ability (Newmark and Sanchez Alvarado, 2002; Elliott and Sanchez Alvarado, 2013). The planarian brain comprises of neurons similar to those found in humans, including dopaminergic, cholinergic, GABAergic, and serotonergic neurons (Nishimura et al., 2007a,b, 2008, 2010). Ca^2+^ signaling plays an important role in neuromuscular signaling and anterior-posterior patterning in planarians regeneration (Nogi et al., 2009). The knockdown of certain voltage-operated Ca^2+^ channels results in different regenerative polarities (Zhang et al., 2011). And knockdown of a specific voltage-operated Ca^2+^ channel (*Ca_*v*_1B*) that impairs muscle function creates an environment permissive for anteriorization in planarian regeneration (Chan et al., 2017). As previously stated, we found that *DjCamKII* and *DjCaln* were both expressed in the brain. Furthermore, *DjCamKII* (RNAi) or *DjCaln* (RNAi) regenerated brains became slim and could not regenerate their lateral nerve. As such, down-regulation of *DjCamKII* or *DjCaln* led to a significant decrease in GAD and affected the number of GABAergic neurons during the planarian head regeneration (Zhen et al., 2020). However, the mechanism behind how *CamKII* and *Caln* regulate the formation of GABAergic neurons during the planarian head regeneration is not clear. The present study aims to examine how Caln, CamkII, and NFAT affect the number of GABAergic neurons during head regeneration in the planarian *Dugesia japonica*.

## Materials and methods

### Animals

A clonal strain of the planarian *D. japonica*, originally obtained from Boshan, China, was established in our laboratory and used in all experiments (Zhen et al., 2020). The *D. japonica* planarians were cultured in Lushan spring water at 20°C. Before use in experiments, all planarians were starved for at least 7 days. For the regeneration experiments, the planarian heads were removed and the fragments were used for experiments at 1, 3, 5, 7, and 10 days after amputation.

### *In situ* hybridization

Whole-mount *in situ* hybridization, digoxigenin (DIG)-labeled antisense RNA probes, and fluorescence *in situ* hybridization (FISH), Fluorescein-labeled antisense RNA probes, were synthesized using an *in vitro* labeling kit (Roche, Basel, Switzerland). The RNA probes-*DjCaln, DjCamKII, DjNFAT*, and *DjGAD* primers were designed by Primer Premier 5.0 (Supplementary Table 1). Hybridizations were carried out by incubation with the antisense RNA probe (1 ng/μl) at 56°C for 16 h. After hybridization, 10% horse serum was blocked at room temperature for 2 h after maleic acid buffer washing. Then, antibodies were diluted in maleic acid buffer containing Tween 20 (MABT) with 10% of horse serum for WISH (anti-DIG-AP, 1:2,000, Roche, Basel, Switzerland) and FISH (anti-DIG-POD, 1:500, and anti-FITC-POD, 1:500, Roche, Basel, Switzerland). Then, a mixture of 5 bromo, 4 chloro, 3-indolyl phosphate, and nitroblue tetrazolium (Roche, Basel, Switzerland) was utilized for color development in WISH, while Cy3-Tyramide and FITC Tyramide (Roche, Basel, Switzerland) [0.001% H_2_O_2_ in PBST (0.01% Tween 20)] was utilized in FISH. The numbers of gad^+^ and TH^+^ neurons in the brain region were counted manually under a confocal microscope (Laser confocal, Leica TCS SP8 MP, Germany) (Hill and Petersen, 2015; Brown et al., 2018). Particles of H3p^+^ and TPH^+^ cells were counted using ImageJ, version 6.0 (National Institutes of Health, United States), and Chat^+^ cells were counted from a zseries of images using three-dimensional segmentation software in Imaris (Hill and Petersen, 2015). Cell counts were statistically analyzed using GraphPad Prism 8 software.

### Ribonucleic acid interference

The template of double-stranded RNA was synthesized by PCR. The dsRNA-*DjCaln, DjCamKII*, and *DjNFAT* primers were designed by Primer Premier 5.0 (Supplementary Table 1). dsRNA was synthesized using MEGAscriptTM RNAi Kit (Invitrogen, United States), and then planarians were soaked in 50 ng/μl dsRNA for 5 h and then transferred to the culture medium (Orii et al., 2003). Control animals were treated with dsRNA of the green fluorescent protein (GFP) (Fraguas et al., 2021).

### Immunohistochemistry

Immunostaining with the mouse monoclonal antibody anti-SYNAPSIN (1:300, 3C11, AB Company, United States) and anti-phosphohistone-H3 (S10) (1:200, Cell Signaling Technology, Danvers, United States) were performed as previously described (Cowles et al., 2012; Lu et al., 2017). First, the planarians were euthanized with 2% of HCl and fixed in paraformaldehyde with phosphate-buffered saline (PBS) at 37°C for 1 h. Then, planarians were dehydrated with 100% of methanol and incubated with the primary antibodies anti-SYNAPSIN and anti-phosphohistone-H3. Incubation with secondary antibodies (1:200, rabbit anti-mouse, and goat anti-rabbit, marked with HRP, Bioss, United States) was performed overnight at 4°C. The samples were then observed using a laser confocal microscope (Leica TCS SP8 MP).

### Western blot

Protein expressions of DjCaln and DjCamkII were quantified with Western blot using the primary antibodies anti-DjCaln and anti-DjCamkII antibodies at 1:500 dilutions. Western blotting was performed as previously described (Gao et al., 2017). Briefly, planarian protein was extracted into PBS buffer, boiled for 20 min, and subjected to SDS-PAGE analysis. After the protein transfer to polyvinylidene fluoride membranes, membranes were blocked with 5% skim milk for 2 h at room temperature (RT). Then, membranes were incubated with primary antibodies (anti-DjCaln and anti-DjCamkII) followed by secondary antibodies (goat anti-rabbit horseradish peroxidase (HRP)-conjugated antibody, 1:5,000, Bioss, United States) overnight at 4°C. Protein signals were observed after treatment with enhanced chemiluminescence solution (Thermo, United States). The quantitation was performed by measuring the optical density of bands with ImageJ, version 6.0 (National Institutes of Health).

### Quantitative real-time PCR

Expressions of *DjCamKII, DjCaln*, and *DjNFAT* mRNA were quantified using qPCR as previously described (Lu et al., 2017). *DjCamKII, DjCaln*, and *DjNFAT* primers were designed by Primer Premier, version 5.0 (Supplementary Table 1). The β*-actin* gene was used as the internal reference. Briefly, total RNA was extracted with TRIol reagent (Invitrogen, United States). The reaction was carried out under the following conditions: initial denaturation at 95°C for 10 s followed by 35 cycles of denaturation at 95°C for 30 s, annealing for 30 s at 55°C, and extension at 72°C for 45 s. The qPCR amplification cycles were performed on the ABI 7,500 real-time PCR system (Applied Biosystems, United States) using the quick-start Universal SYBR Green Master (Roche, Switzerland). Relative expressions compared to β*-actin* were calculated using the 2^–ΔΔct^ method.

### Statistical analysis

Results are presented as mean ± SD. Comparisons between groups were carried out using multiple *t*-tests. For all analyses, *P < 0.05 and ^**^P < 0.01 were considered statistically significant. Statistical analyses were carried out using SPSS statistical software for Windows, version 16.0 (SPSS).

## Results

### Regulatory relationship between DjCaln and DjCamkII during planarian head regeneration

**FIGURE 1:**
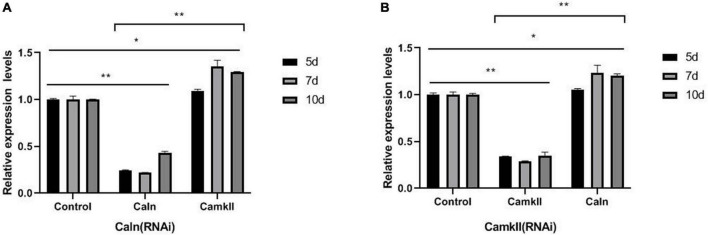
Regulatory relationship between *DjCaln* and *DjCamkII* mRNA during planarian head regeneration. **(A)** *DjCamkII* mRNA levels in *DjCaln* (RNAi) planarians at 5, 7, and 10 days during planarian head regeneration, *n* = 4. **(B)** *DjCaln* mRNA levels in *DjCamkII* (RNAi) planarians at 5, 7, and 10 days during planarian head regeneration, *n* = 4, *p < 0.05, **P < 0.01.

**FIGURE 2:**
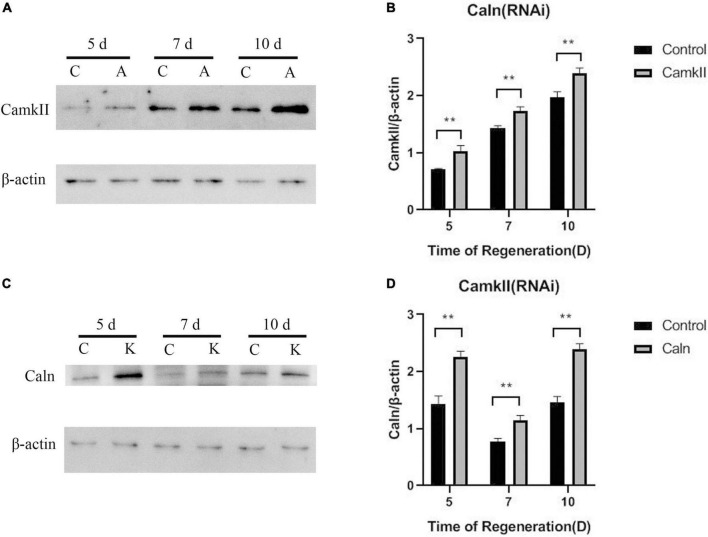
Regulatory relationship between DjCaln and DjCamkII protein during planarian head regeneration. **(A,B)** DjCamkII protein levels in DjCaln (RNAi) planarians at 5, 7, and 10 days during planarian head regeneration, *n* = 5. **(C,D)** DjCaln protein levels in DjCamkII (RNAi) planarians at 5, 7, and 10 days during planarian head regeneration, *n* = 5, **P < 0.01. C, control; A, DjCaln (RNAi); K, DjCamkII (RNAi).

To study the regulation between DjCaln and DjCamkII during planarian regeneration, the mRNA and protein levels of DjCaln and DjCamkII were quantified by qPCR and Western blot. Down-regulation of *DjCaln* led to significant up-regulation of the expression of *DjCamkII* mRNA at 5, 7, and 10 days during planarian head regeneration (Figure 1A). Down-regulation of *DjCamkII* also led to significant up-regulation of the expression of *DjCaln* mRNA at 5, 7, and 10 days of during planarian head regeneration (Figure 1B). Western blot results showed that when the expression of DjCaln protein decreased, the expression of DjCamkII protein significantly increased at 5, 7, and 10 days of during planarian head regeneration (Figures 2A,B). When the expression of DjCamkII protein decreased, the expression of DjCaln protein significantly increased at 5, 7, and 10 days of during planarian head regeneration (Figures 2C,D). These results revealed that DjCamkII and DjCaln were mutual negative regulation during planarian head regeneration.

### *DjNFAT* promotes the expressions of DjCaln and DjCamkII by positive feedback during planarian head regeneration

**FIGURE 3:**
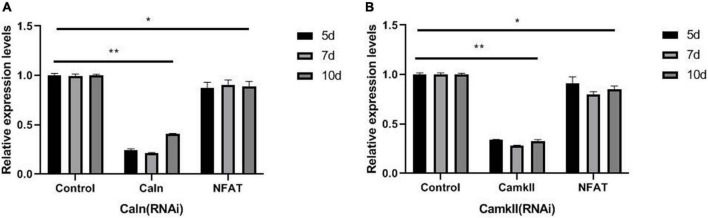
*DjNFAT* mRNA levels in *DjCaln* (RNAi) and *DjCamkII* (RNAi) during planarian head regeneration. **(A)** *DjNFAT* mRNA levels in *DjCaln* (RNAi) planarians at 5, 7, and 10 days during planarian head regeneration, *n* = 4. **(B)** *DjNFAT* mRNA levels in *DjCamkII* (RNAi) planarians at 5, 7, and 10 days during planarian head regeneration, *n* = 4. *p < 0.05, **p < 0.01.

**FIGURE 4:**
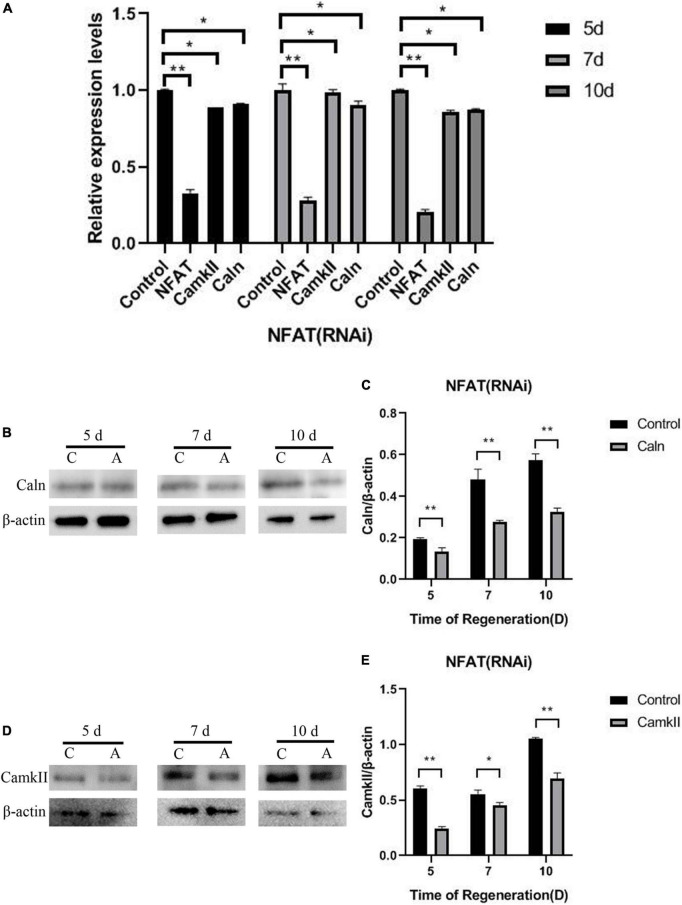
*DjNFAT* regulates *DjCaln* and *DjCamkII* by feedback positive regulation during planarian head regeneration. **(A)** *DjCaln* and *DjCamkII* mRNA level in *DjNFAT* (RNAi) planarians at 5, 7, and 10 days during planarian head regeneration, *n* = 4. **(B,C)** Relative DjCaln protein level in *DjNFAT* (RNAi) planarians at 5, 7, and 10 days during planarian head regeneration, *n* = 5. **(D,E)** Relative DjCamkII protein level in *DjNFAT* (RNAi) planarians at 5, 7, and 10 days during planarian head regeneration, *n* = 5, *P < 0.05, **P < 0.01. C, control; A, DjNFAT (RNAi).

Caln activated NFATs by dephosphorylating multiple N-terminal phosphoserine residues in the regulatory domain (Muller et al., 2009). To explore the relationship between DjCaln, DjCamkII, and DjNFAT, qPCR was performed in *DjCaln* (RNAi), *DjCamkII* (RNAi), or *DjNFAT* (RNAi) regenerative planarians. The results demonstrated that the expression of *DjNFAT* was significantly decreased in *DjCaln* (RNAi) or *DjCamkII* (RNAi) regenerative planarians (Figures 3A,B). Down-regulation of *DjNFAT* led to a significant decrease in the expression of *DjCaln* and *DjCamkII* mRNA (Figure 4A), as well as DjCaln and DjCamkII protein levels (Figures 4B–E) at 5, 7, and 10 days during planarian head regeneration. These results illustrated that *DjNFAT* promoted the expression of *DjCaln* and *DjCamkII* by positive feedback during planarian head regeneration.

### Expression patterns of *DjNFAT* in adult and regenerating planarians

**FIGURE 5:**
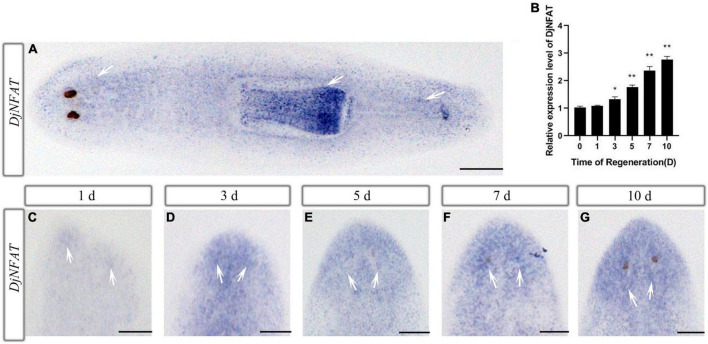
Expression patterns of *DjNFAT* in the intact and regenerating planarians after decapitation. **(A)** Dorsal view of an intact planarian shows an abundant expression in the pharynx and parenchymal cell (arrows). **(B)** qPCR analysis of *DjNFAT* expressions in the regenerating fragments at 0, 1, 3, 5, 7, and 10 days during planarian head regeneration, *n* = 4, *P < 0.05, **P < 0.01. d, day. **(C–G)** WISH showing the expressions of *DjNFAT* in regenerating planarians at 1, 3, 5, 7, and 10 days during planarian head regeneration (arrows), *n* = 6. Scale bars: 0.5 mm.

During the regeneration, *DjCamKII* and *DjCaln* were abundant in the regenerated brain (Zhen et al., 2020). To determine whether *DjNFAT* was involved in brain regeneration, the expression of *DjNFAT* was examined using WISH in decapitated animals as they formed a new brain. The results showed that, in intact planarians, *DjNFAT* was mainly expressed in the pharynx and parenchymal cells (Figure 5A). In head regeneration, at 1 and 3 days into regeneration, the positive signal of *DjNFAT* was predominantly detected at the wound (Figures 5C,D). Then the primordium of the brain continued to change as the regenerated brain began to undergo a pattern formation, in which a positive signal of *DjNFAT* was detected at 5 days of regeneration (Figure 5E). The regeneration of the brain was basically completed at 7 days of regeneration, and the *DjNFAT* positive signal was further strengthened (Figure 5F). At 10 days of regeneration, the *DjNFAT* positive signal of the regenerated brain reached the strongest level, and the regeneration of the lateral nerves was complete (Figure 5G). Using qPCR, changes in the expression of *DjNFAT* mRNA during the head regenerative process were detected, and the results showed that over time, the expression of *DjNFAT* gradually increased, peaking at 10 days of regeneration, which was consistent with the results of WISH (*P* < 0.01) (Figure 5B). In general, the above results indicated that *DjNFAT* was widely expressed in intact and regenerative planarians.

### *DjNFAT* deficiency resulted in brain defects during planarian head regeneration

Trunk fragments of control (RNAi) and *DjNFAT* (RNAi) planarians were stained with anti-phosphohistone H3 (H3P) on days 1, 3, and 5 of regeneration (Brown et al., 2018). The number of dividing cells during regeneration was determined and no significant differences were found between control (RNAi) and *DjNFAT* (RNAi), suggesting that a gross proliferation defect was not present (Supplementary Figure 2). *DjNFAT* (RNAi) regenerating trunk fragments showed a normal regionalized expression of ston2 (Supplementary Figure 1; Zeng et al., 2018), suggesting that *DjNFAT* did not affect neoblast proliferation and brain patterning.

**FIGURE 6:**
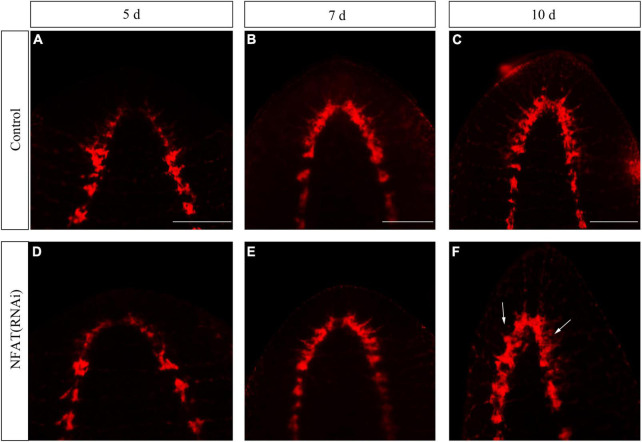
Immunostaining with anti-SYNORF1 in *DjNFAT*(RNAi) treated planarians during planarian head regeneration. **(A–C)** Normal regenerated brains (control), *n* = 5. **(D–F)** The phenotypes of the regenerated brain in the *DjNFAT*(RNAi) treated planarians at 5, 7, and 10 days after amputation, *n* = 6. Anterior is at the front. Scale bars: 0.5 mm.

The knockdown of *DjCamKII* and *DjCaln* led to defects in regenerated brains including incompact phenotypes at the posterior of the new brain, and lateral branches that could not regenerate (Zhen et al., 2020). To study the function of the *DjNFAT* gene in brain regeneration, RNAi was carried out by socking technique commonly used in planarians research. Immunostaining with an anti-SYNORF1 antibody against synapsin (Cebria, 2008) revealed that *DjNFAT*-RNAi treatment caused apparent brain defects at 7 and 10 days of regeneration (Figure 6). Compared to control animals (Figures 6A–C), *DjNFAT*-RNAi regenerated animals were found that the regenerative brain became small and the lateral nerves cannot be regenerated completely (Figures 6D–F). Hence, these results implied that *DjNFAT* was required for the formation of a functional brain during planarian head regeneration.

### *DjNFAT* inhibition influences the number of GABAergic neurons during the planarian head regeneration

To confirm whether or not *DjNFAT* might affect the number of certain types of neurons at 5, 7, and 10 days during neurogenesis, the neurotransmitter synthesizing enzymes were used as the neuron markers in FISH to measure the numbers of neurons in decapitated planarians after *DjNFAT*-RNAi treatment. The results revealed that the numbers of choline acetyltransferase (*DjChat*, cholinergic neurons), tyrosine hydroxylase (*DjTH*, dopaminergic neurons), tryptophan hydroxylase (*DjTPH*, serotonergic neurons) positive-neurons were similar to the controls in the regenerated planarians, suggesting that *DjNFAT*-RNAi could not influence the regeneration of planarian cholinergic, dopaminergic or serotonergic neurons (Supplementary Figure 3).

**FIGURE 7:**
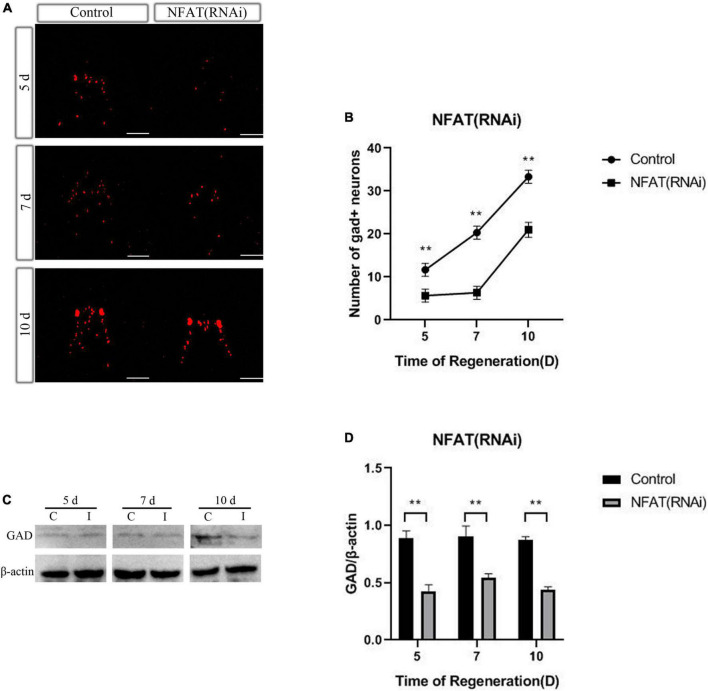
*DjNFAT* inhibition influences the number of GABAergic neurons during planarian head regeneration. **(A)** FISH showing the expression of DjGAD in regenerating planarians at 5, 7, and 10 days after amputation, *n* = 5. Scale bars: 0.2 mm. **(B)** The number of DjGAD-expressing neurons. Error bars represent the SD (**P < 0.01). Anterior is at the front. d, day. **(C)** Western blotting results of DjGAD protein expression in the regenerated head of control and DjNFAT-RNAi planarians, *n* = 4. **(D)** The expression levels of GAD protein in regenerated planarians. C, control; I, *DjNFAT* (RNAi).

As previously presented, knockdown of *DjCamKII* or *DjCaln* led to GABAergic neurons decreased during planarian head regeneration (Zhen et al., 2020). To confirm whether *DjNFAT* also affected the number of GABAergic neurons at 5, 7, and 10 days during neurogenesis, WISH was used to measure the number of GABAergic neurons after *DjNFAT* (RNAi), with the help of neurotransmitter synthase GAD as GABAergic neuron marker. Compared with the control group, the number of DjGAD-positive neurons in planarian regenerated significantly decreased (Figures 7A,B). FISH analysis showed that the number of DjGAD-expressing cells in the newborn brain area was significantly reduced at 5, 7, and 10 days after DjNFAT RNAi (Figure 7B). The expression level of DjGAD protein in *DjNFAT* (RNAi) planarians was detected by Western blot. Compared with control animals, the results showed that DjGAD protein decreased in the regeneration period (Figures 7C,D), which indicated that *DjNFAT* may affect the number of GABAergic neurons during planarian head regeneration through transcriptional regulation of GAD.

## Discussion

The most remarkable characteristic of the planarian resides in its strong regeneration ability. The planarian brain comprises of different types of neurons, including the same type of neurons found in humans, such as dopaminergic, cholinergic, GABAergic, and serotonergic neurons (Nishimura et al., 2007a,b, 2008, 2010). As such, understanding the neural regeneration mechanism of planarians can shed light on how humans make and reintegrate new neurons. As previously explained, down-regulation of *DjCamKII* or *DjCaln* led to the number of GABAergic neurons decreased during planarian head regeneration (Zhen et al., 2020). In this study, the mechanism by which Wnt/Ca^2+^ signaling pathway regulates the formation of GABAergic neurons during planarian neurogenesis was examined. The relationship between DjCamKII and DjCaln suggests a mutual negative regulation during planarian head regeneration. Additionally, knockdown of *DjCaln* or *DjCamKII* reduced the expression of *DjNFAT*, while knockdown of *DjNFAT* could reduce both the mRNA and protein expressions of *DjCaln* and *DjCamKII. DjNFAT* exerted feedback positive regulation on both *DjCaln* and *DjCamkII. DjNFAT* was predominantly expressed in the pharynx and parenchymal cells of the intact planarian, but located in the newborn brain during planarian head regeneration. Down-regulation of *DjNFAT* affected the number of GABAergic neurons and the expression level of DjGAD protein during planarian head regeneration. These findings suggest that the feedback loop between DjCaln, DjCamkII, and DjNFAT is essential for the number of GABAergic neurons during planarian head regeneration.

**FIGURE 8:**
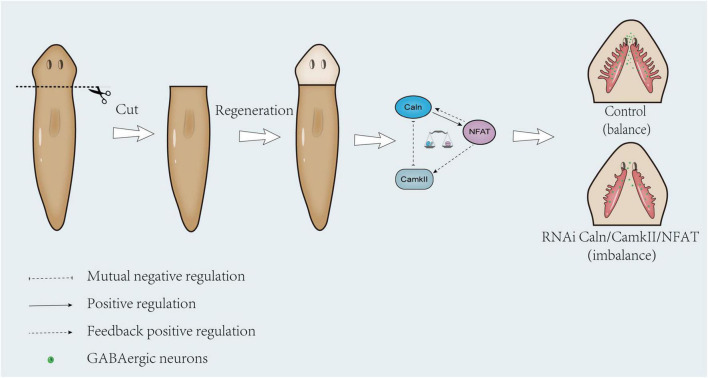
Proposed model illustrating feedback loop between *DjCaln, DjCamkII*, and *DjNFAT* that regulates the number of GABAergic neurons during planarian head regeneration. In the control group, the mutual regulation of *DjCaln, DjCamkII*, and *DjNFAT* was in balance, and the GABAergic neurons could be normal formed during the planarian head regeneration. In the absence of adequate regulation of *DjCaln* or *DjCamkII* after *DjNFAT* RNAi, the number of GABAergic neurons would be reduced during planarian head regeneration.

Previous studies demonstrated that CamKII can mediate phosphorylation of Caln (Hashimoto et al., 1988; Hashimoto and Soderling, 1989; Martensen et al., 1989) and CaMKIIδ_*c*_ can directly phosphorylate Caln to negatively regulate Calcineurin/NFAT signaling in cardiac myocytes (MacDonnell et al., 2009). In budding yeast, the lack of CMK2 up-regulated the level of calcium/calcineurin signaling and augmented the expression levels of Crz1-dependent PMR1 and PMC1 (Xu et al., 2019). In Human T Cells, CaMKII down-regulated both Calcineurin and Protein Kinase C mediated pathways for cytokine gene transcription (Hama et al., 1995). Consistent with previous reports, down-regulation of DjCaln led to increase in the expressions of DjCamkII mRNA and protein at 5, 7, and 10 days during planarian head regeneration. Down-regulation of DjCamkII also led to the increase in the expression of DjCaln mRNA and protein decreased at 5, 7, and 10 days during planarian head regeneration (Figures 1, 2). These results indicated that DjCamKII and DjCaln suggest a mutual negative regulation during planarian head regeneration. Knockdown of *DjCaln* or *DjCamKII* reduced the expression of *DjNFAT*, while the expression of DjCamkII and DjCaln decreased after down-regulation of *DjNFAT* (Figures 3, 4), which was a feedback positive regulation of DjCamkII and DjCaln by DjNFAT. These results indicated that *DjNFAT* exerted feedback positive regulation on both *DjCaln* and *DjCamkII* during planarian head regeneration (Figure 8).

The NFAT protein is part of the family of transcription factors, which usually exists in a hyperphosphorylated state in the cytoplasm (Shaw et al., 1988; Schulz and Yutzey, 2004; Boothby, 2010). The NFATs play a role in many vertebrate developmental systems, including the nervous system (Crabtree and Olson, 2002; Schulz and Yutzey, 2004; Macian, 2005). NFATs (NFATc1–c4) are expressed in neurons (Canellada et al., 2008). For example, NFATc3 and NFATc4 are highly expressed in primary hippocampal neurons (Vihma et al., 2016). The transcription factor Nfat5 was expressed in the anterior blastema of the tail fragments after 3 days into regeneration in planarian *S. mediterranea* (Suzuki-Horiuchi et al., 2021). In contrast to previous studies, in this paper, the results showed that *DjNFAT* was mainly expressed in the pharynx and parenchymal cells in intact planarians (Figure 5A). During planarian head regeneration, *DjNFAT* was located in the blastema at 3 days and perceptible in the brain at 10 days during head regeneration in planarian *D. japonica* (Figures 5C–G). As previously mentioned, *DjNFAT* was difficult to detect in the brain under homeostatic conditions. However, its expression became readily apparent soon after amputation, indicating that *DjNFAT* might participate in newborn brain regeneration.

Studies found that calcineurin and NFAT are essential for neuregulin and ErbB signaling, neural crest diversification, and differentiation of Schwann cells (Kao et al., 2009). Caln/NFAT4 signaling pathway is a key mechanism for the disruption of synaptic remodeling and homeostasis in the hippocampus after acute injury (Furman et al., 2016). Caln/NFAT is also essential in driving glutamate dysregulation and neuronal hyperactivity during AD (Sompol et al., 2017). Moreover, Caln/NFAT signaling plays an important role in the shaping of the synaptic connectivity of thalamocortical and nucleus reticularis thalami GABAergic neurons mediated by slow-wave sleep (Pigeat et al., 2015). As previously described, knockdown of *DjCaln* or *DjCamkII* led to regenerated brains defects including partial deletions and lateral branches not regenerated. And the number of GABAergic neurons also decreased during planarian head regeneration (Zhen et al., 2020). In this study, down-regulation of *DjNFAT* caused defects in regenerated brains including newborn brain small and lateral nerve cannot regenerate (Figure 6). The number of GABAergic neurons in *DjNFAT* (RNAi) planarians was significantly reduced (Figures 7A,B), which is consistent with *DjCaln*-RNAi or *DjCamkII*-RNAi animals (Zhen et al., 2020). In addition, the expression level of DjGAD protein in *DjNFAT* knockdown planarians was down-regulated (Figures 7C,D), which means that *DjNFAT* could regulate the GABAergic neurons during planarian head regeneration. Thus, we conclude that the feedback loop between DjCaln, DjCamkII, and DjNFAT regulates the number of GABAergic neurons during planarian head regeneration (Figure 8).

## Data availability statement

The original contributions presented in this study are included in the article/Supplementary material, further inquiries can be directed to the corresponding author.

## Author contributions

BZ designed this work. BZ and HZ wrote the manuscript. HZ performed the experiments. HZ, MZ, HG, QS, LG, ZY, HD, QP, and BZ analyzed the data. All authors read and approved the final manuscript.

## Supplementary material

Supplementary Figure 1 The expression of DjSton2 in control and DjNFAT(RNAi) regenerated planarians visualized by FISH.

Supplementary Figure 2 Immunostaining of phosphohistone-H3 on days 1, 3, and 5 of regeneration in control and DjNFAT(RNAi) regenerated planarians.

Supplementary Figure 3 The different neuronal types in control and DjNFAT(RNAi) regenerated planarians visualized by FISH. **(A)** FISH of neuronal populations that regenerate properly after DjNFAT(RNAi). Cholinergic neurons (Chat). **(B)** FISH of neuronal populations that regenerate properly after DjNFAT(RNAi). Dopaminergic neurons (TH). **(C)** FISH of neuronal populations that regenerate properly after DjNFAT(RNAi). Serotonergic neurons (TPH). (*n* = 10). Scale bars = 0.2 mm. Anterior is at the front. ns, no significant.
