# Supplementary figures and images for: The feedback loop between calcineurin, calmodulin-dependent protein kinase II, and nuclear factor of activated T-cells regulates the number of GABAergic neurons during planarian head regeneration
Source: Front Mol Neurosci. 2022 Sep 12;15:988803. doi: 10.3389/fnmol.2022.988803 (PMC9510629; doi:10.3389/fnmol.2022.988803)

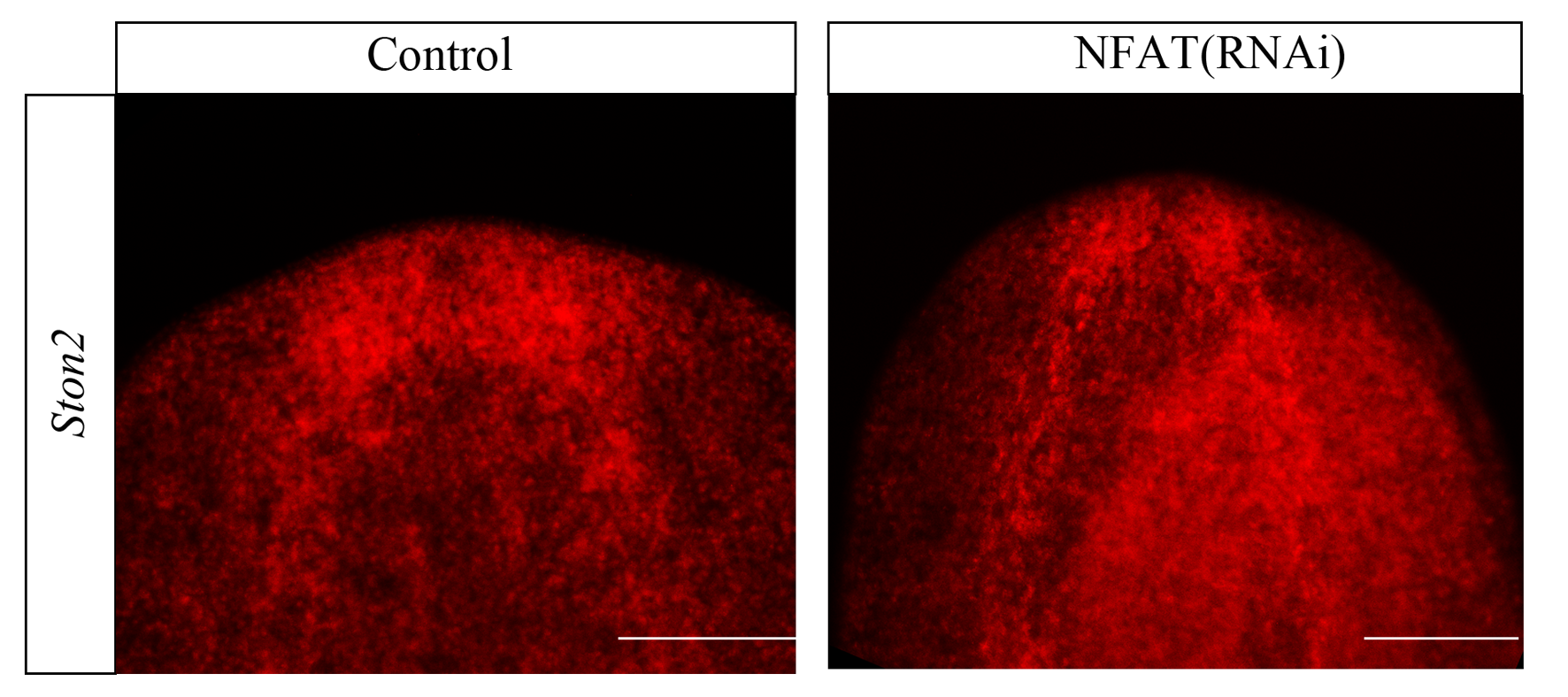

Supplement: Supplementary Figure 1 — The expression of DjSton2 in control and DjNFAT(RNAi) regenerated planarians visualized by FISH. [file Image_1.TIF]

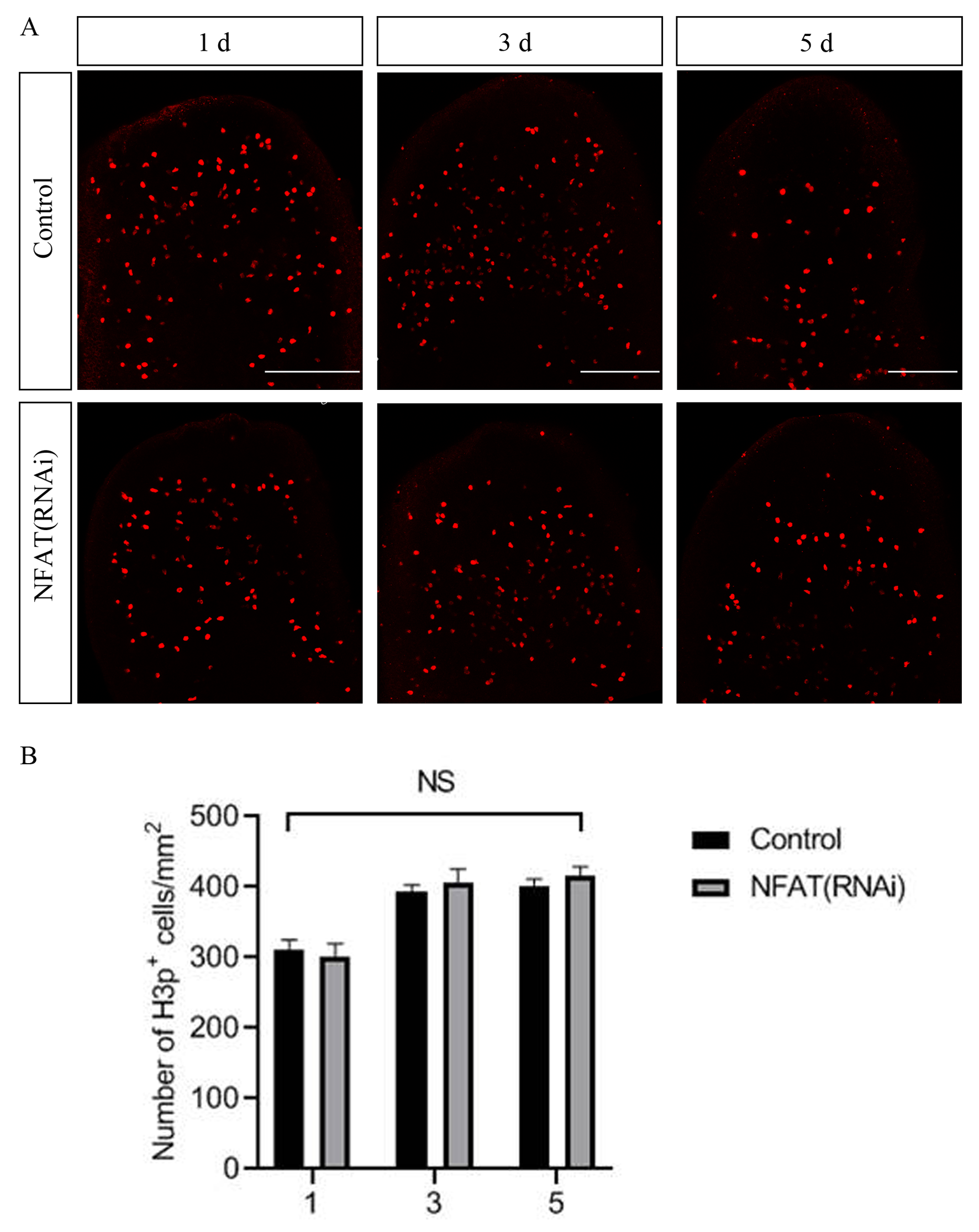

Supplement: Supplementary Figure 2 — Immunostaining of phosphohistone-H3 on days 1, 3, and 5 of regeneration in control and DjNFAT(RNAi) regenerated planarians. [file Image_2.TIF]

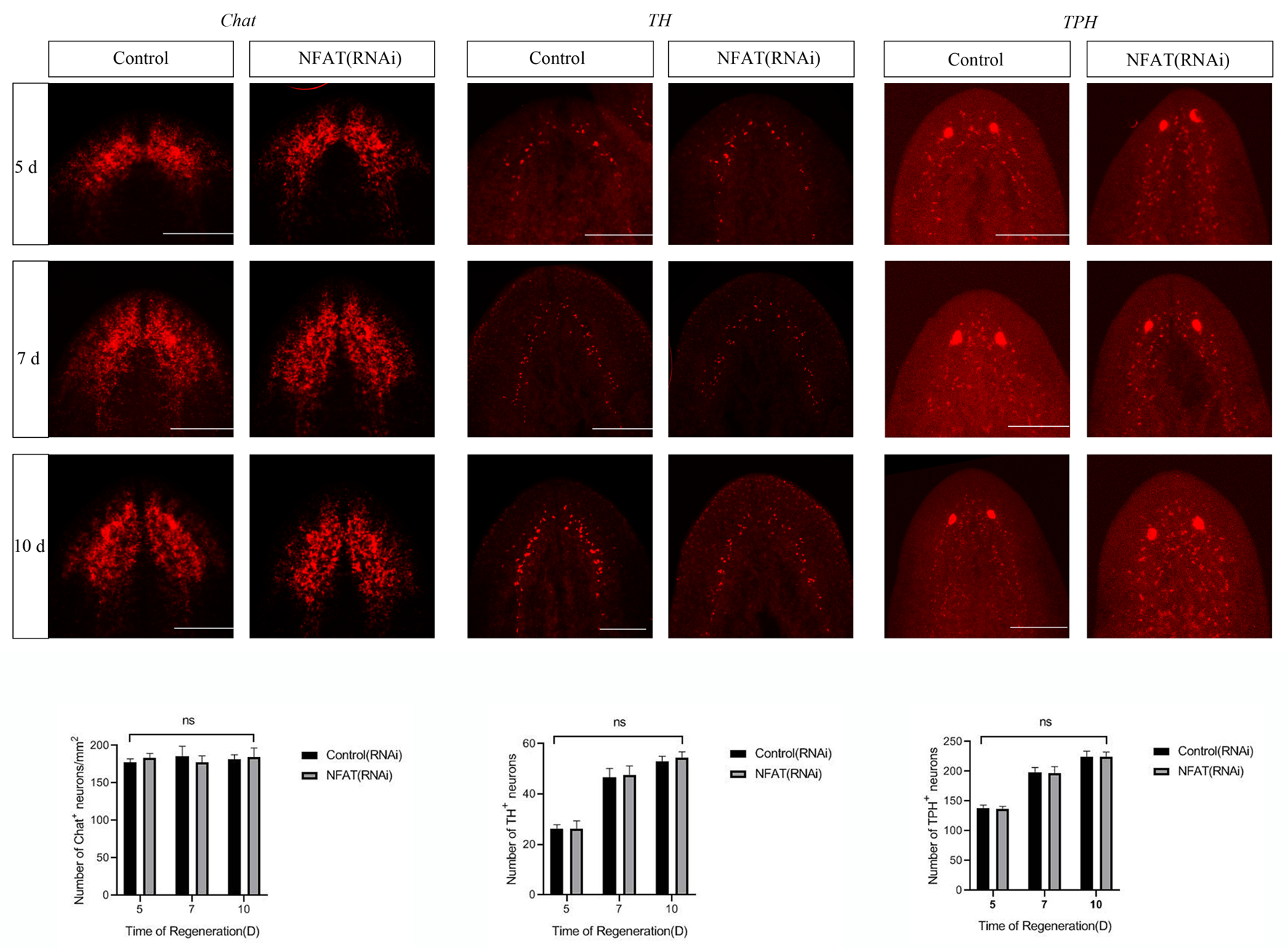

Supplement: Supplementary Figure 3 — The different neuronal types in control and DjNFAT(RNAi) regenerated planarians visualized by FISH. (A) FISH of neuronal populations that regenerate properly after DjNFAT(RNAi). Cholinergic neurons (Chat). (B) FISH of neuronal populations that regenerate properly after DjNFAT(RNAi). Dopaminergic neurons (TH). (C) FISH of neuronal populations that regenerate properly after DjNFAT(RNAi). Serotonergic neurons (TPH). (n = 10). Scale bars = 0.2 mm. Anterior is at the front. ns, no significant. [file Image_3.TIF]
