# Supplementary material for: The feedback loop between calcineurin, calmodulin-dependent protein kinase II, and nuclear factor of activated T-cells regulates the number of GABAergic neurons during planarian head regeneration
Source: Front Mol Neurosci. 2022 Sep 12;15:988803. doi: 10.3389/fnmol.2022.988803 (PMC9510629; doi:10.3389/fnmol.2022.988803)
Supplement: Supplementary file 4 [file Table_1.docx]

Table S1

Primers used in the experiments.

| Name | Forward primer (5’-3’) | Reverse primer (5’-3’) | Size(bp) |
| --- | --- | --- | --- |
| qPCR |  |  |  |
| Caln | TGCCTCAAAATAAACATC | CGTAATAAAGCACCACCT | 208 |
| CamkII | TAAAACTTGTTGACCCTC | TTTACAAATGCAATGGAG | 193 |
| NFAT | CCCGAGTGTTTCTGTAAG | CTATGCTGCTGTTCTGGT | 159 |
| β-actin | ACACCGTACCAATCTATG | GTGAAACTGTAACCTCG | 169 |
|  |  |  |  |
| NFAT |  |  |  |
| Probe | ACCAGAACAGCAGCATAG | GATCACTAATACGACTCACTATAGGGGCATTATCATTGGGTTTC | 981 |
|  |  |  |  |
| dsRNA | GATCACTAATACGACTCACTATAGGGACCAGAACAGCAGCATAG | GATCACTAATACGACTCACTATAGGGGCATTATCATTGGGTTTC | 981 |
| CamkII |  |  |  |
| Probe | GATGAACTAGGCAGAGGT | GATCACTAATACGACTCACTATAGGGTATTGGTTGCGATGCTGT | 997 |
| dsRNA | GATCACTAATACGACTCACTATAGGGGATGAACTAGGCAGAGGT | GATCACTAATACGACTCACTATAGGGTATTGGTTGCGATGCTGT | 997 |
| Caln |  |  |  |
| Probe | TCCTGCTACAACAAGATAC | GATCACTAATACGACTCACTATAGGGATTCCATTCGGAGTGAGT | 975 |
| dsRNA | GATCACTAATACGACTCACTATAGGGTCCTGCTACAACAAGATAC | GATCACTAATACGACTCACTATAGGGATTCCATTCGGAGTGAGT | 975 |
| GAD |  |  |  |
| Probe | ACAGCAATGGCTAATACA | GATCACTAATACGACTCACTATAGGGTTGTACCACTTTCCAGCA | 1030 |
